# The Timely Administration of Epinephrine and Related Factors in Children with Anaphylaxis

**DOI:** 10.3390/jcm11195494

**Published:** 2022-09-20

**Authors:** Lily Myung-Jin Cha, Won Seok Lee, Man Yong Han, Kyung Suk Lee

**Affiliations:** 1 Department of Pediatrics, CHA Ilsan Medical Center, CHA University, Goyang 10414, Korea; 2 Department of Pediatrics, CHA Bundang Medical Center, CHA University, Seongnam 13496, Korea; 3 Department of Pediatrics, Hanyang University Guri Hospital, Hanyang University College of Medicine, Guri 11923, Korea

**Keywords:** anaphylaxis, epinephrine, children, emergency department

## Abstract

Anaphylaxis is a severe allergic reaction that requires immediate recognition and intervention. This study investigated the factors related to the timely administration of epinephrine in cases of pediatric anaphylaxis. We performed a retrospective chart review of 107 patients who visited a pediatric emergency center with anaphylaxis between 2015 and 2017. In total, 76 patients received epinephrine injections. We analyzed factors including allergy history, anaphylaxis signs and symptoms, allergen sensitization, anaphylaxis triggers, and time of epinephrine injection. Anaphylactic patients who received epinephrine took a median of 50 min to arrive at the hospital, and patients who did not receive epinephrine took a median of 94 min. Epinephrine administration was significantly delayed by more than 60 min from symptom onset in patients <2 years old. Patients presenting with wheezing symptoms or history of bronchial asthma were significantly more likely to receive epinephrine within 60 min of symptoms onset, while patients with food allergen sensitization were significantly more likely to receive epinephrine within 30 min of hospital arrival. Wheezing, history of asthma, age (≥2 years old), food triggers, and food allergen sensitivity were significant factors for the rapid administration of epinephrine. An immediate diagnosis of anaphylaxis and a rapid administration of epinephrine are essential.

## 1. Introduction

Anaphylaxis is an acute allergic reaction that can cause life-threatening symptoms at any time from a few minutes to a few hours after exposure [1]. Various clinical symptoms involving the cutaneous, respiratory, cardiovascular, and gastrointestinal systems can be observed [2]. The median time to a potential respiratory or cardiac arrest in food-related anaphylaxis was found in one study to be 30 min [3]. Because anaphylaxis can affect multiple organs in a short time, immediate recognition and intervention are crucial [4]. 

In 2006, approximately 2% of the global population experienced anaphylaxis [5]. Recent epidemiologic studies have reported a worldwide annual incidence of between 50 and 112 episodes per 100,000 individuals, and the estimated lifetime prevalence more than doubled [6]. The annual incidence of anaphylaxis in children ranges between 1 and 761 episodes per 100,000, a number that has approximately tripled since 1998 [7]. The diagnosis of anaphylaxis is based on the recognition of characteristic symptoms that occur after exposure to a potential causative trigger [8]. The most common cause of anaphylaxis in children is a food allergy, with cow’s milk and eggs the most frequent triggers [6].

Current guidelines recommend epinephrine administration as the first-line agent in the treatment of anaphylaxis [1,2]. Other medications such as corticosteroids, antihistamines, and inhalable medications can also be used, but epinephrine remains the gold standard [9]. Alpha-1 adrenergic effects decrease mucosal edema and beta-1 adrenergic effects increase the rate and force of cardiac contractions, preventing hypotension and shock, while beta-2 adrenergic effects increase bronchodilation and decrease inflammatory mediator release from mast cells and basophils [9].

Although the prevalence of anaphylaxis is increasing worldwide, treatment often remains inadequate [10]. Unfortunately, anaphylaxis is not easily recognized because of the inconsistent clinical symptoms. Moreover, even when it is correctly diagnosed, epinephrine administration is often delayed [11]. Various studies have shown that the delayed administration of epinephrine leads to higher rates of hospitalization [12] and fatalities [13]. Prompt epinephrine administration, therefore, is universally emphasized, although studies regarding the specific timing of epinephrine administration are limited. Therefore, this study aimed to investigate the factors related to the timely administration of epinephrine in pediatric anaphylaxis. 

## 2. Materials and Methods

### 2.1. Patients

We evaluated the medical charts of 80,981 patients who visited a single pediatric emergency center between 1 January 2015, and 31 December 2017. This pediatric emergency department is a vital medical care center that concentrates specifically on the needs of children under 15 years of age in the local community. Upon review, 146 of the patients had an anaphylactic incident that could be classified as either T78.0, T78.2, or T88.6 under the International Classification of Disease (ICD)-10 codes. From these initially recruited study subjects, 107 children who met the diagnostic criteria for anaphylaxis based on the National Institutes of Health symposium [1] were included in the final study population and underwent a retrospective medical record review by pediatricians. This study was approved by the Institutional Review Board (IRB) of CHA University Bundang CHA Hospital (IRB number 2018-04-023).

### 2.2. Patient Characteristics, Severities of Anaphylaxis, Allergen Sensitization Status, and Administration Time of Epinephrine

Clinical characteristics of sex, age, history of allergic disease, family history of allergic disease, signs and symptoms, specific allergen sensitization, epinephrine injection time from hospital arrival, epinephrine injection time from symptom onset, and hospital admissions were all reviewed. Allergic diseases associated with anaphylaxis, such as bronchial asthma, drug allergies, food allergies, allergic rhinitis, and atopic dermatitis were evaluated in each patient’s history and familial allergy history. 

The signs and symptoms used in our analysis were urticarial rash, facial edema, throat tightness, dyspnea (described subjectively by the patient or caregiver), wheezing (observed by a physician), hypoxemia (oxygen saturation < 92%), vomiting, abdominal pain, syncope, and hypotension (systolic blood pressure <70 mm Hg + (2 × age in years) in children aged 1–10 years, <90 mm Hg in children ≥10 years of age) [2,14,15].

No overall consensus exists on which system can accurately grade the severity of anaphylaxis. The modifications of the World Allergy Organization’s (WAO) grading system were originally designed to classify systemic allergic reactions (SARs) for use with allergen immunotherapy. However, the system has been adapted for all SARs regardless of cause [1,2,16]. In this classification, some grades, such as 3, 4, and 5, would be consistent with the definition of anaphylaxis [1,2,16]. A Grade 3 SAR is defined as the presence of lower airway symptoms (e.g., mild bronchospasm, cough, wheezing, and shortness of breath, which respond to treatment), gastrointestinal symptoms (e.g., abdominal cramps, vomiting, diarrhea), and uterine cramps [1,2,16]. A Grade 4 SAR is defined as the presence of lower airway symptoms (e.g., severe bronchospasm, not responding, or worsening, with treatment) and upper airway symptoms (e.g., laryngeal edema with stridor) [1,2,16]. A Grade 5 SAR is defined as the presence of lower or upper airway symptoms (e.g., respiratory failure), cardiovascular symptoms (e.g., collapse, hypotension), and loss of consciousness (except vasovagal causes) [1,2,16].

ImmunoCAP (Thermo Fisher, Uppsala, Sweden), MAST (AdvanSure AlloScreen, Seoul, South Korea), and skin prick test (SPT) were all used to evaluate a patient’s allergen sensitization status. The majority of ImmunoCAP and MAST tests were conducted while patients were being treated for anaphylaxis in the emergency department, although a few ImmunoCAP and MAST tests, and all of the SPTs, were conducted at the outpatient clinic after management in the emergency department. The definition of a positive specific allergen followed the definition of previous studies [17,18].

Evaluating the administration time of epinephrine was approached in two ways. First, patients were divided depending on whether their epinephrine injection time from hospital arrival was either <30 min or ≥30 min. Second, patients were divided depending on whether their epinephrine injection time was either <60 min or ≥60 min from symptom onset. The onset time of the anaphylaxis symptoms was based upon statements given either by the patients or their caregivers. 

### 2.3. Statistical Analysis

The results were statistically analyzed using the Mann–Whitney U test for continuous variables and the chi-squared test for nominal variables. A logistic regression analysis was conducted to identify the risk factors for rapid epinephrine injection in anaphylaxis patients, and *p*-values < 0.05 were considered statistically significant. All data were analyzed using SPSS software version 26.0 (IBM, Armonk, NY, USA).

## 3. Results

### 3.1. Clinical Characteristics of Subjects 

**Figure 1:**
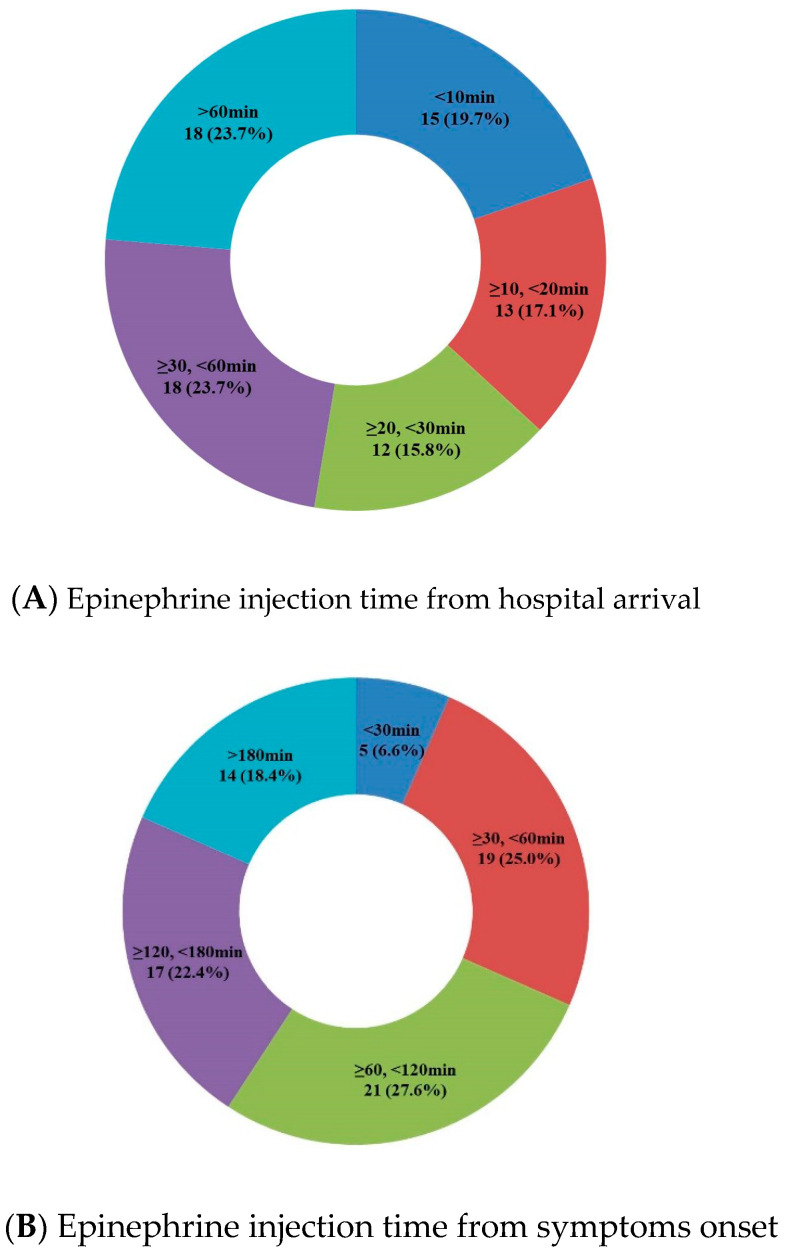
Injection times from hospital arrival and symptom onset. (**A**) Epinephrine was administered within 30 min of hospital arrival for 52.6% of the patients, while the remaining 47.4% received epinephrine after 30 min or more. (**B**) Epinephrine was administered within 60 min of symptoms onset for 31.6% of than patients and more than 60 min for the remaining 68.4%.

The clinical characteristics of the participants are summarized in Table 1. Among the patients with anaphylaxis, 76 (71%) were injected with epinephrine, compared with 31 (29.0%) who were not. The reasons why epinephrine was not administered were because some patients’ symptoms significantly improved before arriving at the emergency room (ER); other subjects took several hours to arrive at ER with symptom relief; and some patients received treatment for anaphylaxis at other clinics or hospitals before being transferred to the ER. Of the 76 patients who received epinephrine injections, 42 (55.3%) were male, with a median age of 3.5 years (interquartile range 1.0–7.8 years). Most patients, 57 (75.0%), had a history of allergies, while 26 (34.2%) had a familial history of allergies. None of the patients was diagnosed with either mast cell disease or mastocytosis. The median arrival time at the hospital was 50.0 min (interquartile range 29.3–104.8 min). Detailed epinephrine injection times from hospital arrival and symptoms onset are shown in Figure 1. Of the 31 patients who did not receive epinephrine, 21 (67.7%) were male with a median age of 5.0 years (interquartile range 1.0–7.8 years). Additionally, 50 (64.5%) patients had a history of allergies, and 10 (32.3%) had a familial history of allergies. The median arrival time for these patients at the hospital was 94.0 min (interquartile range 42.5–146.5 min). There was a statistically significant difference in the arrival time at the hospital between the two groups.

### 3.2. Comparison of the Causes, Symptoms, Treatment, and Outcomes between the Group Who Received Epinephrine and the Group That Did Not

Among the 76 patients who received epinephrine, 68 (89.5%) presented with urticarial rash, 51 (67.1%) had facial edema, 50 (65.8%) had dyspnea, 29 (38.2%) had gastrointestinal symptoms (nausea, vomiting, or abdominal pain), 27 (35.5%) had wheezing, 10 (13.2%) had throat tightness, 7 (9.2%) had syncope, and 4 (5.3%) had hypotension; additionally, 1 patient (1.3%), who was triggered by traditional herbal medicine, presented with hypotension but no skin rashes. Additionally, 56 patients (73.7%) were classified as either Grade 3 or 4 SAR, while 20 patients (26.3%) had a Grade 5 SAR. The triggers causing anaphylaxis were food in 46 patients (60.5%), immune treatment in 7 (9.2%), drugs in 5 (6.6%), exercise in 1 (1.3%), and unknown in the remaining 17 patients (58.6%). Next, 49 patients (60.5%) showed allergen sensitization, of whom 37 (66.1%) showed food allergen sensitization and 28 (50%) showed inhalant allergen sensitization. In addition to epinephrine, 67 patients (88.2%) were given steroids, 66 patients (86.8%) were given antihistamines, and 17 patients (22.4%) had nebulization therapy. Finally, 13 patients (17.1%) had been prescribed self-injectable epinephrine when discharging from ER or at an outpatient clinic after their discharge from ER. After treatment in the ER, 24 patients (31.6%) were admitted to the hospital, while 52 (68.4%) were discharged (Table 2).

Among the 31 patients who did not receive epinephrine, 24 (77.4%) presented with urticarial rash, 23 (74.2%) had dyspnea, 17 (54.8%) had facial edema, 10 (32.3%) had gastrointestinal symptoms (nausea, vomiting, or abdominal pain), 7 (22.6%) had wheezing, 5 (16.1%) had throat tightness, 5 (9.7%) had hypotension, and 2 (6.5%) had syncope. One patient presented with hypotension without skin features (3.23%), and one patient had syncope without skin features (3.23%). By SAR grade, 26 patients (83.9%) were classified as either Grade 3 or 4 SAR, while the remaining 5 (16.1%) had a Grade 5 SAR. The triggers that caused anaphylaxis were food in 12 patients (38.7%), drugs in 3 patients (9.7%), exercise in 2 patients (6.5%), immune treatment in 1 patient (3.2%), and unknown in the remaining 12 patients (38.7%). Furthermore, 15 patients (78.9%) showed allergen sensitization, of whom 11 (57.9%) showed food allergen sensitization and 8 (4.1%) showed inhalant allergen sensitization. In addition to epinephrine, 26 patients (83.9%) received steroids, 23 patients (74.2%) received antihistamines, and 5 (16.1%) received nebulization therapy. Finally, 5 of the patients (16.1%) were prescribed self-injectable epinephrine either when discharging from the ER or at an outpatient clinic after discharge from the ER. After treatment in the ER, 4 patients (12.9%) were admitted to the hospital, while the remaining 27 (87.1%) were discharged (Table 2). None of the 107 patients’ anaphylaxis was caused by either contrast medium or anesthetics.

### 3.3. Comparison of the Injection Time of Epinephrine from Hospital Arrival and Symptom Onset 

The epinephrine injection time from hospital time or symptom onset divided by age group is shown in Table 3. Patients younger than 2 were significantly more likely to be given epinephrine within 60 min (*p* = 0.015). There were no statistically significant differences among the other age groups. 

An analysis of the comparison of epinephrine injection time according to various factors is shown in Table 4. Patients with a history of bronchial asthma (*p* = 0.016), food triggers (*p* = 0.022), and immune treatment triggers (*p* = 0.001) were significantly more likely to be given epinephrine within 30 min of hospital arrival.

The results of the logistic regression show the analysis of the conditions associated with epinephrine injection within 30 min of hospital arrival and 60 min of symptoms onset. Patients presenting with wheezing (adjusted odd ratio (aOR) 3.209, 95% CI 1.005–10.244, *p* = 0.049) or a history of bronchial asthma (aOR 3.605, 95% CI 1.104–11.766, *p* = 0.034) were significantly more likely to be given epinephrine within 60 min of symptoms onset. Additionally, patients with food allergen sensitization (aOR 3.990, 95% CI 1.026–15.518, *p* = 0.046) were significantly more likely to be given epinephrine within 30 min of hospital arrival (Table 5).

## 4. Discussion

This study evaluated clinical characteristics such as allergic history, triggers, signs and symptoms, allergen sensitization status, and emergency treatments of anaphylactic patients who presented at a pediatric emergency department with anaphylaxis and assessed the administration time of epinephrine from hospital arrival and symptom onset. To the best of our knowledge, this is the first study to investigate the factors related to the timely administration of epinephrine in cases of pediatric anaphylaxis.

Anaphylaxis is an acute, potentially life-threatening allergic reaction where the rapid administration of epinephrine can be a life-saving treatment [19]. Treatment guidelines recommend that 0.01 mg/kg (maximum of 0.5 mg) of epinephrine be delivered by intramuscular injection into the lateral thigh [19]. However, studies show a lack of consistency among treatment guidelines for epinephrine injection, particularly with regard to pediatric patients [20]. Several studies have reported that approximately 50% of patients receive epinephrine treatment in cases of pediatric anaphylaxis [21], although a Korean study reported that only 24% of patients received proper treatment [20].

In our study, 71% of the patients received epinephrine, which was a higher value than in other studies in the literature. This could be because the medical staff members at this renowned pediatric specialist center were able to make prompt decisions about the patients’ anaphylaxis treatment. Furthermore, our study shows that patients presenting with wheezing symptoms or an allergic history of bronchial asthma were significantly more likely to receive epinephrine within 60 min of symptoms onset, while patients with food allergen sensitization ere significantly more likely to receive epinephrine within 30 min of hospital arrival. However, any causal relationships are unknown at present.

It is well known that food is the most common trigger of pediatric anaphylaxis [6,22]. The most common food allergens are cow’s milk in infants, peanuts in children, and tree nuts and shellfish in young adults [22]. Peanuts and tree nuts have been identified as being primarily responsible for the majority of fatal anaphylaxis cases in children [23]. According to our previous study, children under two years of age were more sensitized to food allergens than to inhalant allergens [24], and there were significant associations between anaphylaxis symptoms and allergen sensitization [24]. Facial edema is associated with patients sensitized to food allergens, while wheezing is associated with patients sensitized to milk, wheat, nuts, and crustaceans. Additionally, vomiting is associated with food sensitization and milk allergens. In other words, these results show that children with food allergen sensitization are more likely to develop wheezing, facial edema, and vomiting symptoms during anaphylaxis, although more research is needed to clarify the association between food allergen sensitization and the timely administration of epinephrine in anaphylaxis.

According to a recent study, bronchial asthma is an independent risk factor for severe anaphylaxis [25]. Another study reported that having a history of asthma is strongly associated with severe anaphylaxis in children, especially in food-related anaphylaxis [26]. Our study showed that epinephrine is more likely to be administrated quickly in anaphylactic children with a history of bronchial asthma.

A 10-year study conducted in the US showed that the incidence of food-related anaphylaxis has increased in children of all ages, with the highest increases reported in infants aged 0 to 2 years [7]. A multicenter study conducted in Korea, for example, shows that the number of cases of anaphylaxis in infants aged 0 to 2 nearly quadrupled between 2009 and 2013 [27].

Despite the increased numbers of infantile anaphylaxis cases, our study shows a significantly delayed administration of epinephrine in the 0 to 2 age group. This could be because meaningful communication with children under two years old is nearly impossible and since they cannot make their own decisions, the responsibility falls upon the parents. Since it can be difficult for parents to recognize anaphylaxis symptoms in their infant children, it may take them longer to realize that their child is seriously ill and to travel to the emergency department. There are also difficulties in physically examining very young children. Listening for auscultation can be particularly challenging as the child is likely to be crying. All these factors can prolong the time it takes for physicians to diagnose anaphylaxis and immediately administer epinephrine.

The timely administration of epinephrine is important in pediatric anaphylaxis since it can prevent hospitalization and fatalities [12,28]. However, the lack of a standardized protocol, low awareness of guidelines, and concern about side effects can all cause delays in epinephrine administration [19]. The potential adverse effects of epinephrine, such as palpitations, tremors, and anxiety, are more prevalent in adults but are usually transient and rarely require specific treatment [19]. Since anaphylaxis symptoms in children may be unclear and nonspecific, parents and caregivers should increase their vigilance, particularly if the child has had a previous episode of anaphylaxis. Previous anaphylaxis history is an important risk factor for subsequent severe anaphylaxis in children and adults, particularly if they have suffered reactions to bee stings and drugs [29]. Parents and caregivers should also look out for anaphylaxis symptoms, have an action plan for anaphylactic emergencies, and to learn how to use self-injectable epinephrine [30]. Physicians in emergency departments should also be alert to potential signs of anaphylaxis in young children, especially those under two years of age, and administer a timely epinephrine dose.

Our study has several limitations. A causal relationship was difficult to prove due to the retrospective design, plus the number of enrolled patients was relatively small. Furthermore, if a child with anaphylaxis arrived relatively late to the emergency department with improved symptoms, the physician would be more likely to decide not to administer epinephrine. However, we did include and analyze the symptoms both before arrival at the hospital and at the time of hospital arrival. After considering all the symptoms both before and after hospital arrival, the physician would determine the severity of the anaphylaxis and would decide on treatment, which may or may not include epinephrine. We set the epinephrine administration time as 30 min from arrival at the hospital and 60 min from symptom onset, but this is a subjective criterion as the critical time for epinephrine administration cannot be clearly defined. More importantly, it should be emphasized that epinephrine must be administered as soon as possible if a patient has unmistakable anaphylactic symptoms. However, this study did attempt a meaningful investigation into the factors related to the timely administration of epinephrine in pediatric anaphylactic patients.

In conclusion, pediatric patients presenting with wheezing, a history of allergic asthma, food triggers, and food allergen sensitization receive a significantly rapid dose of epinephrine. As the incidence of anaphylaxis in children, particularly infants, is increasing rapidly, prompt recognition and treatment are vital. Future studies regarding the specific timing of the epinephrine injection may increase awareness and help to deliver timely epinephrine treatment, thereby improving the prognosis of pediatric anaphylactic patients.

## Figures and Tables

**Table 1:** Characteristics of the subjects (epinephrine injected group vs. non-injected group) (n = 107).

	Epinephrine Injected (n = 76)		Epinephrine not Injected (n = 31)		Total (n = 107)		*p*-Value
	n	%	n	%	n	%	
Sex (boy, n,%)	42	55.30%	21	67.70%	63	58.90%	0.234
Age (median, yr) †	3.5 (1.0–7.75)		5.0 (3.0–10.0)		4.0 (1.0–8.0)		
Age group							0.212
<2 yr	24	31.60%	5	16.10%	29	27.10%	
≥2, <6 yr	26	34.20%	11	35.50%	37	34.60%	
≥6 yr	26	34.20%	15	48.40%	41	38.30%	
All allergic histories	57	75%	20	64.5	77	72%	0.273
Anaphylaxis	8	10.5	2	6.5	10	9.3	0.511
Bronchial asthma	16	21.1	3	9.7	19	17.8	0.162
Drug allergy	4	5.3	1	3.2	5	4.7	0.651
Food allergy	31	40.8	11	35.5	42	39.3	0.610
Allergic rhinitis	21	27.6	6	19.4	27	25.2	0.371
Atopic dermatitis	27	35.5	7	22.6	34	31.8	0.192
Familial allergy history	26	34.2	10	32.3	36	33.6	0.846
Arrival time to hospital (median, min) †	50.0 (29.25–104.75) (n = 76)		94 (42.5–146.5), (n = 29)				<0.001 *
Eosinophil (median, %)	1.0 (0.6–2.1), (n = 55)		1.0 (0.65–3.65), (n = 21)		1.0 (0.625–2.25), (n = 76)		0.378
Total IgE (U/mL)	146 (42.5–420.5), (n = 54)		121 (62.5–304.0), (n = 21)		132.9 (47.9–380), (n = 75)		0.648

* indicates a *p*-value of < 0.05, and † indicates the interquartile range.

**Table 2:** Comparison of epinephrine administration (injected group vs. not injected group) with anaphylaxis signs and symptoms, severity, triggers, allergen sensitization status, treatments, and hospital admissions (n = 107).

		Epinephrine Injected (n = 76)		Epinephrine not Injected (n = 31)		Total		*p*-Value
		n	%	n	%	n	%	
Anaphylaxis symptoms and signs	Urticarial rash	68	89.5	24	77.4	92	86	0.103
	Facial edema	51	67.1	17	54.8	68	63.6	0.232
	Throat tightness	10	13.2	5	16.1	15	14	0.688
	Dyspnea	50	65.8	23	74.2	73	68.2	0.093
	Wheezing	27	35.5	7	22.6	34	31.8	0.192
	Gastrointestinal symptoms	29	38.2	10	32.3	39	36.4	0.565
	Syncope	7	9.2	2	6.5	9	8.4	0.641
	Hypotension	4	5.3	3	9.7	7	6.5	0.402
Anaphylaxis severity in definition of SAR	Grade 3,4	56	73.7	26	83.9	82	76.6	0.259
	Grade 5	20	26.3	5	16.1	25	22.4	0.259
Anaphylaxis trigger	Food	46	60.5	12	38.7	58	54.2	0.040 *
	Immune treatment	7	9.2	1	3.2	8	7.5	0.286
	Exercise	1	1.3	2	6.5	3	2.8	0.144
	Drug	5	6.6	3	9.7	8	7.5	0.580
	Unknown	17	58.6	12	38.7	29	27.1	0.085
Allergen sensitization	All allergens	49	87.5	15	78.9	64	85.3	0.363
	Inhalant allergens	28	50	8	4.1	36	48	0.552
	Food allergens	37	66.1	11	57.9	48	64	0.521
Treatments	Antihistamine	66	86.8	23	74.2	89	83.2	0.113
	Steroid	67	88.2	26	83.9	93	86.9	0.551
	Nebulization therapy ^a^	17	22.4	5	16.1	22	20.6	0.469
	Self-injectable epinephrine	13	17.1	5	16.1	18	16.8	0.903
Treatment results	Admission to hospital	24	31.6	4	12.9	28	26.2	0.046 *

Abbreviations: SAR, Systemic allergic reaction; * indicates a *p*-value of <0.05.; Nebulization therapy^a^ indicates any drug administration with nebulized element can be used in children, following medications are included: beta-2 agonist, corticosteroids, and epinephrine.

**Table 3:** Comparison of the epinephrine injection time from hospital arrival (<30 min vs. ≥30 min) and symptom onset (<60 min vs. ≥60 min), divided by age group (n = 76).

	Epinephrine Injection Time from Hospital Arrival						Epinephrine Injection Time from Symptom Onset					
	Total (n = 76), (Median, min) †	<30 min, (n = 40)		≥30 min, (n = 36)		*p*-Value	Total (n = 76) (Median, min) †	<60 min (n = 24)		≥60 min (n = 52)		*p*-Value
		n	%	n	%			n	%	n	%	
Age group												
<2 yr (n = 24, 31.6%)	39.5 (19.25–69.5)	9	37.5	15	62.5	0.730	120.5 (69.5–148.75)	3	12.5	21	87.5	0.015 *
≥2, <6 yr (n = 26, 34.2%)	25.5 (13.0–49.0)	16	61.5	10	38.5	0.262	69.5 (42.0–133.0)	11	42.3	15	57.7	0.147
≥6 yr (n = 26, 34.2%)	23.0 (8.75–51.5)	15	57.7	11	42.3	0.524	77.0 (42.75–152.25)	10	38.5	16	61.5	0.352
Total (n = 76)	28.0 (12.50–57.50)	40	100	36	100		89.5 (49.25–143.75)	24	100	52	100	

* indicates a *p*-value of < 0.05, and † indicates the interquartile range.

**Table 4:** Comparison of the groups divided by epinephrine injection time from hospital arrival (< 30 min vs. ≥30 min) and symptom onset (<60 min vs. ≥60 min) (n = 76).

		Epinephrine Injection Time from Hospital Arrival					Epinephrine Injection Time from Symptoms Onset				
		<30 min, (n = 40)		≥30 min, (n = 36)		*p*-Value	<60 min, (n = 24)		≥60 min, (n = 52)		*p*-Value
		n	%	n	%		n	%	n	%	
Allergic history	All allergic history	33	82.5	24	66.7	0.111	21	87.5	36	69.2	0.087
	Anaphylaxis	5	12.5	3	8.3	0.555	3	12.5	5	9.6	0.703
	Bronchial asthma	11	27.5	5	13.9	0.146	9	39.5	7	13.5	0.017 *
	Drug allergy	4	10	0	0	0.051	3	12.5	1	1.9	0.055
	Food allergy	18	45	13	36.1	0.431	11	45.8	20	38.5	0.543
	Allergic rhinitis	12	30	9	25	0.626	7	29.2	14	26.9	0.839
	Atopic dermatitis	16	40	11	30.6	0.390	10	41.7	17	32.7	0.447
Familial allergy history		14	35	12	33.3	0.878	9	37.5	17	32.7	0.681
Anaphylaxis symptoms and signs	Urticarial rash	35	51.5	33	48.5	0.555	20	29.4	48	70.6	0.236
	Facial edema	28	54.9	23	45.1	0.571	16	31.4	35	68.6	0.956
	Throat tightness	4	40	6	60	0.391	1	10	9	90	0.115
	Dyspnea	27	54	23	46	0.740	16	32	34	68	0.913
	Wheezing	15	55.6	12	44.4	0.705	11	40.7	16	59.3	0.202
	Gastrointestinal symptoms	13	44.8	16	55.2	0.284	7	24.1	22	75.9	0.273
	Hypotension	1	25	3	75	0.255	0	0	4	100	0.163
	Syncope	3	42.9	4	57.1	0.587	2	28.6	5	71.4	0.857
Grade 5 SAR (vs. Grades 3&4 SAR)		10	50.0	10	50.0	0.800	6	30.0	14	70.0	1.000
Triggers	Food	23	50	23	50	0.569	10	21.7	36	78.3	0.022 *
	Immune treatment	6	85.7	1	14.3	0.066	6	85.7	1	14.3	0.001 *
	Exercise	1	100	0	0	0.340	1	100	0	0	0.138
	Drug	2	40	3	60	0.558	2	40	3	60	0.675
	Unknown	8	47.1	9	52.9	0.601	5	29.4	12	70.6	0.827
Allergen sensitization	All allergen sensitization	30	75	19	52.8	0.113	17	70.8	32	61.5	0.546
	Inhalant allergen	15	37.5	13	36.1	0.364	11	45.8	17	32.7	0.508
	Food allergen	24	60	13	36.1	0.114	12	50	25	48.1	0.982
Treatments other than epinephrine	Antihistamine	33	50	33	50	0.238	19	28.8	47	71.2	0.179
	Systemic corticosteroid	34	50.7	33	49.3	0.369	18	26.9	49	73.1	0.016 *
	Nebulizer	11	64.7	6	35.3	0.258	5	29.4	12	70.6	0.827
	Self-injectable epinephrine	7	53.8	6	46.2	0.923	5	38.5	8	61.5	0.558
Treatment results	Admission to hospital	13	54.2	11	45.8	0.856	7	29.2	17	70.8	0.759

Abbreviations: SAR, Systemic allergic reaction; * indicates a *p*-value of < 0.05.

**Table 5:** Logistic regression analysis for the conditions associated with epinephrine injection within 30 min of hospital arrival (vs. ≥30 min) and within 60 min of symptoms onset (vs. ≥60 min) (n = 76).

A. Anaphylaxis Symptoms and Signs								
	Epinephrine Injected Time from Hospital Arrival <30 min, (n = 40)				Epinephrine Injected Time from Symptom Onset <60 min (n = 24)			
	cOR	*p*-Value	aOR ^a^	*p*-Value	cOR	*p*-Value	aOR ^a^	*p*-Value
Urticarial rash	0.636 (0.141–2.876)	0.557	1.312 (0.211–8.143)	0.771	0.417 (0.095–1.832)	0.247	0.810 (0.127–5.161)	0.823
Facial edema	1.319 (0.505–3.441)	0.572	1.635 (0.56–4.698)	0.362	0.971 (0.348–2.715)	0.956	1.310 (0.414-–0.142)	0.646
Throat tightness	0.556 (0.143–2.153)	0.395	0.572 (0.131–2.491)	0.457	0.208 (0.025–1.743)	0.148	0.122 (0.011–1.342)	0.085
Dyspnea	1.174 (0.455–3.032)	0.740	1.316 (0.467–3.710)	0.603	1.059 (0.381–2.945)	0.913	1.183 (0.376–3.719)	1.183
Wheezing	1.200 (0.467–3.082)	0.705	1.337 (0.491–3.645)	0.570	1.904 (0.703–5.153)	0.205	3.209 (1.005–10.244)	0.049 *
Hypotension	3.545 (0.352–35.724)	0.283	3.311 (0.313–34.968)	0.320				
Gastrointestinal symptoms	0.602 (0.237–1.530)	0.286	0.659 (0.236–1.837)	0.425	0.561 (0.199–1.585)	0.276	0.582 (0.178–1.898)	0.369
Syncope	1.542 (0.321–7.410)	0.589	1.525 (0.310–7.510)	0.604	1.170 (0.210–6.510)	0.858	1.123 (0.184–6.855)	0.900
**B. Allergic history**								
	**Epinephrine Injected Time from Hospital Arrival <30 min, (n = 40)**				**Epinephrine Injected Time from Symptom Onset <60 min (n = 24)**			
	**cOR**	***p*-Value**	**aOR ^b^**	***p*-Value**	**cOR**	***p*-Value**	**aOR ^b^**	***p*-Value**
Previous anaphylaxis	1.571 (0.348–7.101)	0.557	1.408 (0.301–6.585)	0.664	1.343 (0.293–6.146)	0.704	0.949 (0.190–4.741)	0.949
Bronchial asthma	2.352 (0.728–7.593)	0.153	2.248 (0.688–7.342)	0.180	3.857 (1.224–12.153)	0.021 *	3.605 (1.104–11.766)	0.034 *
Drug allergy	-	-	-	-	7.286 (0.716–74.102)	0.093	6.636 (0.597–73.760)	0.124
Food allergy	1.448 (0.576–3.641)	0.432	1.516 (0.582–3.951)	0.394	1.354 (0.509–3.601)	0.544	1.561 (0.533–4.575)	0.417
Allergic rhinitis	1.286 (0.467–3.541)	0.627	1.147 (0.367–3.586)	0.814	1.118 (0.382–3.266)	0.839	0.647 (0.176–2.381)	0.512
Atopic dermatitis	1.515 (0.586–3.919)	0.391	1.503 (0.574–3.933)	0.407	1.471 (0.543–3.986)	0.448	1.450 (0.508–4.137)	0.488
**C. Allergen Sensitization Status**								
	**Epinephrine Injected Time from Hospital Arrival <30 min, (n = 40)**				**Epinephrine Injected Time from Symptoms Onset <60 min (n = 24)**			
	**cOR**	***p*-Value**	**aOR ^b^**	***p*-Value**	**cOR**	***p*-Value**	**aOR ^b^**	***p*-Value**
All allergen sensitization ^c^	3.947 (0.695–22.436)	0.121	4.047 (0.680–24.081)	0.124	3.187 (0.354–28.687)	0.301	2.433 (0.254–23.427)	0.442
Inhalant allergen sensitization	0.747 (0.258–2.158)	0.590	0.466 (0.120–1.803)	0.268	1.941 (0.619–6.089)	0.255	1.019 (0.231–4.484)	0.980
Food allergen sensitization	2.538 (0.817–7.886)	0.107	3.990 (1.026–15.518)	0.046 *	1.040 (0.317–3.409)	0.948	1.876 (0.462–7.617)	0.379

Abbreviations: cOR crude odd ratio; aOR, adjusted odd ratio.; * indicates a *p*-value of < 0.05.; ^a^ adjusted by sex, age, familial allergy history, severity of anaphylaxis (severe vs. mild to moderate), anaphylaxis history, allergic rhinitis, and drug allergy history.; ^b^ adjusted by sex, age, and familial allergy history.; ^c^ All allergen sensitization: at least one of the allergy tests is positive for any allergens, and allergens include both inhalant and food allergen, except drugs, Hymenoptera venom, etc.

## Data Availability

The datasets used and analyzed during the current study are available from the corresponding author upon reasonable request.
